# Hepatitis C virus and HIV co-infection among pregnant women in Rwanda

**DOI:** 10.1186/s12879-017-2269-0

**Published:** 2017-02-22

**Authors:** Mwumvaneza Mutagoma, Helene Balisanga, Dieudonné Sebuhoro, Aimable Mbituyumuremyi, Eric Remera, Samuel S. Malamba, David J. Riedel, Sabin Nsanzimana

**Affiliations:** 1 Rwanda Biomedical Center, Ministry of Health, P.O. Box: 7162, Kigali, Rwanda; 2 US Centers for Disease Control and Prevention (CDC), Center for Global Health (CGH), Division of Global HIV/AIDS (DGHA), Kigali, Rwanda; 3 Institute of Human Virology and Division of Infectious Diseases, University of Maryland School of Medicine, Baltimore, MD USA

**Keywords:** Hepatitis C virus, HIV, Pregnant women, HIV-HCV co-infection

## Abstract

**Background:**

Hepatitis C virus (HCV) infection is a pandemic causing disease; more than 185 million people are infected worldwide. An HCV antibody (Ab) prevalence of 6.0% was estimated in Central African countries. The study aimed at providing HCV prevalence estimates among pregnant women in Rwanda.

**Methods:**

HCV surveillance through antibody screening test among pregnant women attending antenatal clinics was performed in 30 HIV sentinel surveillance sites in Rwanda.

**Results:**

Among 12,903 pregnant women tested at antenatal clinics, 335 (2.6% [95% Confidence Interval 2.32–2.87]) tested positive for HCV Ab. The prevalence of HCV Ab in women aged 25–49 years was 2.8% compared to 2.4% in women aged 15–24 years (aOR = 1.3; [1.05–1.59]); This proportion was 2.7% [2.37–2.94] in pregnant women in engaged in non-salaried employment compared to 1.2% [0.24–2.14] in those engaged in salaried employment (aOR = 3.2; [1.60–6.58]). The proportion of HCV Ab-positive co-infected with HIV was estimated at 3.9% (13 cases). Women in urban residence were more likely to be associated with HCV-infection (OR = 1.3; 95%CI [1.0–1.6]) compared to those living in rural setting.

**Conclusion:**

HCV is a public health problem in pregnant women in Rwanda. Few pregnant women were co-infected with HCV and HIV. Living in urban setting was more likely to associate pregnant women with HCV infection.

## Background

The global prevalence of hepatitis C virus (HCV) infection was estimated between 1.2 and 3.8% according to World Health Organization (WHO) Global Burden Disease (GBD), with more than 185 million people infected worldwide [1–4]. The region with the highest reported HCV prevalence in the world was found in Central Asia (3.8%). In sub-Saharan Africa, the prevalence was 2.3% [1, 5].

A study conducted in specific sub-Saharan African countries estimated a high prevalence of HCV (6.0%) in central African countries. In North Africa, Egypt has the highest HCV prevalence (17.5%) followed by Morocco (7.7%) [6, 7].

In Rwanda, HCV seroprevalence studies have been limited to HIV- infected persons only [8, 9]. A study conducted among adults in one HIV clinic in the capital, Kigali in 2010, showed that the prevalence of HCV antibody (anti-HCV) was 5.7% [8]. The second found a sero-prevalence of 2.1% among HIV-infected persons in Rwanda [9].

Sexual and maternal to child transmission and household exposure are not listed among major factors of HCV transmission [2, 6]. The transmission of HCV from mother to child is estimated at 4–8%, but the transmission rate increases to 17–25% if the mother is also HIV infected [10]. In sub-Saharan Africa the main factor of HCV prevalence is blood transfusions and unsterile injections [6].

The prevalence of HCV infection among pregnant women was estimated between 1 and 8% worldwide [11]. In the Arabian Peninsula and North African (Maghreb) region, HCV among pregnant women ranges from 8.6% in Egypt to less than 1% in Libya [12]. In Nigeria, a study conducted among pregnant women, found that the prevalence of HCV ranged between 1.4% for women aged between 20 and 35 years old and 11.1% for women aged less than 20 years old [13]. In a systematic review and meta-analysis study published in 2015, conducted in 21 sub-Saharan African countries, found that the overall HCV prevalence among pregnant women in antenatal clinic was 3% and was 2% in the Central African region [14].

The burden of HCV infection in the general population and among pregnant women is unknown in Rwanda. The objective of the current study in Rwanda was to determine the prevalence and factors associated with testing positive for anti-HCV among pregnant women attending antenatal care services.

## Methods

### Study design

In 2011, an HIV cross-sectional sero-surveillance activity included HCV antibody testing among other tests performed in pregnant women from June 2011 to December 2011. A signed informed consent was used.

### Setting and sites

In Rwanda before 2005, only 24 sentinel sites participated in HIV surveillance. In order to be more representative, the number was increased to 30 sentinel sites in 2005, with 14 located in urban areas and 16 in rural areas. Each province has at least two urban and two rural sites. Inclusion criteria for sites were: at least 80 new women enrolled for ANC services per month, geographically accessible, at least one midwife or a nurse experienced in maternity or ANC, and one laboratory technician. Hepatitis C screening was added in the 2011 survey.

### Sentinel population

All pregnant women aged 15–49 years presenting for the first time for their current pregnancy for antenatal clinic and prevention of mother-to-child transmission (ANC-PMTCT) services during the data collection period and voluntarily agreeing to a venous blood draw for HIV and syphilis testing routine testing, were enrolled in the survey. Personal socio-demographic characteristics were collected using a surveillance form that was complete through interview conducted by trained data collectors who were also health center regular staff. The option of linking personal data was used to return hepatitis results to participants.

Women younger than 18 years were accompanied by an adult guardian and signed assent form to allow the participation in the survey. A sample size of 13,267 will result in a relative 10% precision around a point estimate (HIV prevalence) of 3% among pregnant women attending ANC at an alpha of 0.05 and power of 0.80.

### Laboratory methods

#### Hepatitis C virus testing

All hepatitis C screening tests were performed at the central level at the National Reference Laboratory (NRL) in Kigali. Hepatitis C antibody (HCV Ab) using Abbott ARCHITECT system included, Reagents, calibrator, control packs, Pre-Trigger Solution and Probe Conditioning Solution for the assays for screening for prior exposure to hepatitis C and/or current infection.

#### HIV testing

HIV testing was performed at the health facility on all eligible and consenting pregnant women samples using 3 rapid test algorithm in order to provide without delay HIV test result to participants for counseling and clinical management for HIV-positive according to the national testing guidelines.

For the survey quality assurance purpose, all positive and 10% of the negative were sent to NRL for retesting using ELISA-based HIV testing algorithm.

### Data collection

Data collectors were selected among health facility staff. At each health facility, focal persons, including the Director and one laboratory technician, were selected to carry out data collection. Five-day training was organized to familiarize staff with the protocol, laboratory techniques related to the surveillance, and standard operating procedures (SOPs). Participants were consecutively enrolled according to the inclusion criteria until the determined sample size at the site was reached. A structured sero-surveillance questionnaire was administered to all eligible pregnant women attending ANC/PMTCT service. Forms were kept in a secure place at the sentinel sites and transported to the Rwanda Biomedical Center (RBC) after every 2 weeks during regular site supervision visits. Questionnaires and laboratory results were double-entered using Epi Info (CDC, Atlanta, GA) at RBC.

### Data analysis

STATA software (StataCorp LP, 4905 Lakeway Drive, College Station, TX, USA) was used for data analysis. HCV prevalence, HCV-HIV co-infection proportions and 95% confidence limits were estimated separately by levels of socio-demographic factors. Bivariate logistic regression analysis was used to test for associations between screened HCV infection and potential risk factors. The multivariate analysis was performed to determine factors that were associated with HCV infection in the bivariate analysis at the ≤0.1 significance level to develop the final multi-variable model using a backward elimination method. All variables were modeled as categorical variables to check on potential interactions and associations. None of the tested interaction terms between age and marital status and age and education was significant. The prevalence of screened HCV, among pregnant women was estimated at 95% confidence intervals. The risk factors for HCV infection among pregnant women attending ANC services were determined from covariates fitted in the multi-variable logistic regression analysis: age, marital status, education level, occupation, residence, parity and, HIV infection.

### Ethical considerations

In 2011, blood specimen and interview-based data collection was only performed on volunteers eligible participants who provided informed consent. The survey was reviewed in accordance with CDC human subjects review procedures and was determined to be a non-research routine disease surveillance activity. The survey was approved by the Rwandan National Ethics Committee.

## Results

Of, 13,292 pregnant women enrolled in the survey, 63.8% were 25 years old and above, 87.2% were married or cohabitating and 88.6% were illiterate. By source of income, 96.1% were not salaried. A proportion of 39.5% of women were at their 2–3 parity, 30.7% were at their first parity. Among all pregnant women, 12,903 (97.1%) were screened for HCV Ab, 335 (2.6%) [95%CI: 2.3–2.9] were identified HCV Ab-positive.

The prevalence of HCV-screened positive was 2.4% [95% CI: 1.6–3.2] among single pregnant women, 2.7% [95% CI: 2.3–2.9] among married pregnant women, and 3.9% [95% CI: 1.2–6.5] among separated, divorced or widowed pregnant women. The prevalence of HCV-screened positive was higher in pregnant women with low education level (illiterate and primary education level) compared to those with high education level (secondary and university education level): 2.7% [95% CI: 2.4–3.0] for illiterate pregnant women or pregnant women with primary education level vs. 1.8% [95%CI: 2.0–2.4 with secondary or university level]. Prevalence of HCV-screened positive was higher among pregnant women in unsalaried employment compared to pregnant women in salaried employment with 2.7% [95%CI: 2.4–3.0] and 1.2% [95%CI: 0.2–2.1] respectively (Table 1)

**Table 1:** Hepatitis C virus and HIV co-infection among pregnant women attending ante-natal clinics in Rwanda, 2011

Characteristics	Total	Hepatitis C prevalence			HIV co-infection proportion among HCV Ab- positive		
	N	n	%	[95% C.I]	n	%	[95% C.I]
Age group							
15–24	4,669	109	2.3	[1.91–2.77]	1	0.9	[-0.78–2.38]
25–49 yrs	8,160	226	2.8	[2.41–3.13]	12	5.3	[1.76–6.19]
Marital status							
Single	1,438	35	2.4	[1.64–3.23]	1	2.9	[-1.94–5.78]
Married/Cohabiting	11,166	291	2.7	[2.32–2.92]	11	3.8	[1.30–4.94]
Divorced/Separated/Widow	207	8	3.9	[1.22–6.51]	1	12.5	[-5.47–15.47]
Education							
Illiterate/Primary	11,319	305	2.7	[2.40–2.99]	12	3.9	[1.40–4.99]
Secondary/University	1,468	26	1.8	[1.96–2.45]	1	3.8	[-2.25–6.55]
Employment							
Salaried(Salaried employee + Housemaids)	505	6	1.2	[0.24–2.14]	1	16.7	[-26.18–59.60]
Non-salaried	12,268	326	2.7	[2.37–2.94]	12	3.7	[1.27–4.49]
Residence							
Rural	6,416	151	2.4	[1.98–2.73]	4	2.6	[0.77–6.76]
Urban	6,487	184	2.8	[2.43–3.25]	9	4.9	[1.17–4.75]
Number of Pregnancies							
1 Pregnancy^a^	3,886	100	2.6	[2.75–3.72]	0	0.0	
2-3 pregnancies	4,944	130	2.6	[2.18–3.76]	8	6.2	[1.25–6.67]
4-5 pregnancies	2,384	61	2.6	[1.92–3.19]	4	6.6	[0.84–7.26]
> = 6 pregnancies	1,356	38	2.8	[1.92–3.68]	1	2.6	[-3.62–1.51]
HIV status							
Negative	12,463	322	2.6	[2.36–2.86]	-	-	-
Positive	429	13	3.0	[1.42–4.66]	13	100	
Total	12,903	335	2.6	[2.32–2.87]	13	3.9	[1.40–4.66]

^a^Including the current one

Note: The denominator for co-infection is HCV Ab-positive

HIV infection among HCV Ab positive was analyzed. Out of 335 screened HCV Ab-positive, 13 women were co-infected with HIV. The proportion of HIV infection among HCV-infected women was estimated at 3.9% [95% CI: 1.4–4.7]. This co-infection among HCV infected women varied in the different socio-demographic characteristics of pregnant women attending ANC (Table 1).

Education level, employment and residence were considered in determining factors associated with HCV infection (Table 2). In multivariate analysis, there is evidence that HCV among pregnant women was associated with urban residence with aOR = 1.3 [95% CI: 1.0–1.6].

**Table 2:** Hepatitis C Virus infection associated factors among pregnant women attending ante-natal clinics in Rwanda, 2011

	Bivariate			Multivariable		
	cOR	*p*-value	[95% CI]	aOR	*p*-value	[95% CI]
Age group						
15–24 years						
25–49 years	1.19	0.14	[0.95,1.50]	-		
Education						
Illiterate/primary						
Secondary/University	0.65	0.04	[0.43,0.98]	0.69	0.10	[0.45,1.07]
Marital status						
Single						
Married/cohabiting	1.07	0.70	[0.75,1.53]	-		
Divorced/Separated/Widow	1.61	0.23	[0.74,3.52]	-		
Employment						
Salaried						
Non-salaried		0.04	[1.00,5.12]	1.87	0.15	[0.80,4.40]
Residence						
Rural						
Urban	1.21	0.09	[0.97,1.51]	1.28	0.03	[1.02,1.60]
Pregnancies						
1 Pregnancy						
2-3 pregnancies	1.02	0.87	[0.79,1.33]	-		
4-5 Pregnancies	0.99	0.97	[0.72,1.37]	-		
> = 6 Pregnancies	1.09	0.65	[0.75,1.59]	-		
HIV status						
Negative						
Positive	1.18	0.57	[0.67,2.07]	-		

NB: If the bivariate *p*-value was >0.1 – variable was not included in multivariable analysis

## Discussion

All attendees to ANC services participated in the study and all consented for HCV testing. The overall prevalence of HCV Ab screened positive among pregnant women in sentinel sites in Rwanda was estimated at 2.6%. Due to insufficient blood sample, 2.9% of participants were not tested for HCV. Pregnant women aged between 25 and 49 years were more likely to be infected with HCV than younger pregnant women (15–24 years old). The proportion of HIV-positive among HCV Ab-positive pregnant women in ANC sentinel surveillance sites was (3.9%).

The HCV prevalence among pregnant women in sentinel sites in Rwanda was similar to the overall HCV prevalence in sub-Sahara African countries (3.0%) and the prevalence of HCV in Central African region (2.0%) [13]. It was also similar to the prevalence using confirmed HCV-positive tests in Malawi (2.0%) [15], and higher than the HCV prevalence among pregnant women in Brazil, United Kingdom (London) and Nigeria of 0.2, 0.8 and 1.5% respectively [12, 15]. In the African region, the prevalence of HCV in the general population widely varied by country, ranging from 0.1 to 17.5% [7]. The increase of HCV with age was observed in a study conducted in Brazil among pregnant women in 2009 [16] and in all African regions [9]. A study of a global epidemiology of hepatitis C virus infection found that HCV prevalence increased with age with different HCV prevalence peak in different ages in different regions [1]. The HCV and HIV co-infection proportion among pregnant women attending the ANC services in Rwanda was 3.9%.

The HCV and HIV co-infection was low among HCV-positive pregnant women in Rwanda. The proportion of the co-infection was lower compared to published studies in sub-Sahara countries (16.0%) and in Central African region (6.0%). A Brazilian study published in 2009 reported 7.0% of HCV-positive pregnant women were co-infected with HIV-1 [15]. In European countries, among HIV-positive enrolled pregnant women this co-infection was very high (12.3%) [17]. In 2010, a meta-analysis [15] focusing on HIV and HCV co-infection using published studies in consecutive years from several sub-Saharan African countries for adult people published the following results: In 2006 HCV and HIV co-infection was 4.8% in Burkina Faso and in 2007, it was 8.6% in Cameroon. In 2008 HCV and HIV co-infection in Ethiopia was 1.8%; 1.8% in Kenya, 1.1% in Malawi, 15.7% in Mozambique, 8.0% in Senegal, 13.4% in South Africa and 18.1% in Tanzania. In another study, it was reported that HCV and HIV co-infection in Nigeria was 3.8% in 2009 [14].

A significant difference in odds of having HCV was observed among pregnant women in urban residence compared to rural residence. That is, the HCV risk was higher in the urban than the rural women. The assumption behind this situation is the usual location of key population in urban setting than in rural ones.

In our study, there was no evidence to suggest a significant association between HIV and HCV infections. Similar observations were made in a study conducted in Kigali among adult HIV-positive persons [8].

The co-infection of HIV and HCV is associated with high mortality due to liver diseases compared to mono-infection [18]. Therefore, HIV is considered an important risk factor for HCV in the general population but has not been widely investigated. Most published studies on HCV risk factors were conducted among high-risk groups such as sex workers, injecting drug users and men who have sex with men and blood transfusion [2, 6].

This study had several limitations, namely: The survey was part of the antenatal sentinel sites sero-surveillance and therefore findings are applied to pregnant women in sentinel sites and cannot be generalized to the whole population in Rwanda or to pregnant women in other sub-Saharan African countries. Secondly, due to the small number of HCV and HIV co-infected pregnant women in our survey, it was not possible to assess the magnitude of this co-infection in selected socio-demographic characteristics sub-groups. Thirdly, in the present survey, Abbott ARCHITECT system is a screening test with high sensitivity. Therefore, in the context of our study, this screening test that was originally used in blood transfusion services to minimize hepatitis C transmission from blood donors; we may have some false HCV Ab-positive results.

## Conclusion

HCV-infection is a public health burden among pregnant women in Rwanda. This study provides a platform to inform policymakers and stakeholders the need to accelerate progress in treatment access and global prevention of HCV infections.

Further advanced studies of HCV in this group and in the general population are needed to explore detection of Antigens and Antibodies and how they inform the diagnosis of acute and active HCV infections.

## Abbreviations

Ab: Antibody
AIDS: Acquired Immunodeficiency Syndrome
ANC: Antenatal care
aOR: Adjusted Odds Ratio
CDC: Centre for disease control and prevention
CI: Confidence interval
ELISA: Enzyme-Linked Immuno-Sorbent Assay
GBD: Global Burden Disease
HBV: Hepatitis B Virus
HCV: Hepatitis C Virus
HIV: Human immunodeficiency virus
NRL: National Reference Laboratory
OR: Odds ratio
PEPFAR: President’s Emergency Plan for AIDS Relief
PMTCT: Prevention of mother to child transmission
RBC: Rwanda Biomedical Center
SOPs: Standard Operating Procedures
WHO: World Health Organization
